# Predictive value of chemotherapy-induced neutropenia for the efficacy of oral fluoropyrimidine S-1 in advanced gastric carcinoma

**DOI:** 10.1038/sj.bjc.6603831

**Published:** 2007-06-05

**Authors:** T Yamanaka, S Matsumoto, S Teramukai, R Ishiwata, Y Nagai, M Fukushima

**Affiliations:** 1 Cancer Biostatistics Laboratory, Institute for Clinical Research, National Kyushu Cancer Centre, 3-1-1 Notame, Minami-ku, Fukuoka 811-1395, Japan; 2 Department of Translational Clinical Oncology, Graduate School of Medicine, Kyoto University, Kyoto 606-8507, Japan; 3 Department of Clinical Trial Design and Management, Graduate School of Medicine, Kyoto University, Kyoto 606-8507, Japan; 4 Translational Research Informatics Centre, Kobe 650-0047, Japan

**Keywords:** gastric cancer, S-1, neutropenia, prognosis, time-dependent variable

## Abstract

Myelosuppression that occurs during chemotherapy has been reported to be a predictor of better survival in patients with breast or lung carcinomas. We evaluated the prognostic implications of chemotherapy-induced neutropenia in advanced gastric carcinoma. Data from a prospective survey of oral fluoropyrimidine S-1 for advanced gastric cancer patients in Japan were reviewed. We identified 1055 untreated patients with adequate baseline bone marrow function. During treatment with S-1, a total of 293 (28%) patients experienced grade 1 or higher neutropenia. The adjusted hazard ratio of death for the presence of neutropenia, as compared with the absence of such toxicity, from a multivariate Cox model was 0.72 (95% confidence interval, 0.54–0.95; *P*=0.0189) for grade 1 neutropenia, 0.63 (0.50–0.78; *P*<0.0001) for grade 2 neutropenia and 0.71 (0.51–0.98; *P*=0.0388) for grade 3–4 neutropenia. These findings suggest that the occurrence of neutropenia during chemotherapy is an independent predictor of increased survival in patients with advanced gastric cancer, whereas the absence of such toxicity indicates that the dosages of drugs are not pharmacologically adequate. Monitoring of neutropenia in patients who receive chemotherapy may contribute to improved drug efficacy and favourable survival.

The dose intensity is recognised as a key element in a patient’s response to cytotoxic drugs. In general, it is considered that the higher the dose intensity, the greater the chance that the optimal dose is approached (Frei and Canellos, 1980). Neutropenia is one of the most important dose-limiting toxicities of cytotoxic drugs, often necessitating a reduction from the initial dosage (Crawford *et al*, 2004). Therefore, many oncologists may consider that the absence of haematological toxicity will raise the hope of an adequate tumour response without myelosuppression. Since the late 1990s, however, several studies have reported that neutropenia or leucopenia occurring during chemotherapy is a sign of patient’s response to adjuvant chemotherapy in women with breast cancer; a significantly longer survival results in patients who have those haematological toxic effects (Saarto *et al*, 1997; Poikonen *et al*, 1999; Mayers *et al*, 2001; Cameron *et al*, 2003). A recent study by Di Maio *et al* (2005) confirmed the positive correlation between chemotherapy-induced neutropenia and increased survival in a pooled analysis of three randomised trials that included a total of 1265 patients with advanced non-small-cell lung cancer (NSCLC). Consideration of these findings led us to review data from a prospective survey of Japanese patients with advanced gastric cancer, and to investigate the association between neutropenia occurring during chemotherapy and patient survival. Our goal was to provide the initial evidence, through a rigorous statistical analysis, in a large series of subjects with advanced gastric cancer as to the utility of neutrophil count as a surrogate indicator of drug efficacy.

## MATERIALS AND METHODS

### Patients

The subjects of this study were identified from among the patients participating in a nationwide survey of oral fluoropyrimidine derivative S-1 (TS-1®; Taiho Pharmaceutical Co. Ltd, Tokyo, Japan) in Japan (Shirasaka *et al*, 1996). This survey was prospectively conducted by the manufacturer to obtain safety data of the drug, and all patients in Japan scheduled for S-1 administration were centrally registered from March 1999 through March 2000. The protocol of this survey was approved by all participating centres, and informed consent was obtained from all the patients prior to participation in this study.

A total of 3758 patients with advanced gastric cancer were enrolled at more than 700 centres. We identified 1055 subjects who met the following criteria: age less than 80 years, Eastern Cooperative Oncology Group (ECOG) performance status (PS) of 0 or 1, sufficient bone marrow function (neutrophils ⩾2.0 × 10^9^ l^−1^, leucocytes ⩾4.0 and ⩽12.0 × 10^9^ l^−1^, platelets ⩾100 × 10^9^ l^−1^ and haemoglobin ⩾9.5 g dl^−1^) and no history of chemotherapy within 6 months before the commencement of S-1 treatment. The patients were followed up until March 2002 to obtain survival information.

### Treatment delivery

S-1 was to be taken twice a day orally after meals. The initial dose was assigned based on the body surface area (BSA) as follows: BSA <1.25 m^2^, 40 mg (80 mg day^−1^); BSA ⩾1.25 and <1.50 m^2^, 50 mg (100 mg day^−1^); BSA ⩾1.50 m^2^, 60 mg (120 mg day^−1^). A single therapy cycle consisted of S-1 monotherapy for 28 consecutive days, followed by 14 days of no treatment. This schedule was repeated every 6 weeks unless the disease progressed or unacceptable adverse effects occurred. The dose modification scheme followed that in the previous trials of S-1 (Sakata *et al*, 1998; Koizumi *et al*, 2000); when haematological adverse reactions at grade 3–4 or non-haematological reactions at grade 2–4 appeared, the dose was reduced from 120 to 100 mg day^−1^ and from 100 to 80 mg day^−1^, respectively, or administration was temporarily discontinued.

The dose intensity was calculated as the total dose administered divided by the duration of time over which it was given. The relative dose intensity (RDI) was then calculated as the ratio of the actual dose intensity to the ideal value if planned doses were all given on schedule (Hryniuk and Levine, 1986).

### Study endpoint and assessment of haematological toxicity

The study endpoint was overall survival. This was defined as the interval between the date of the beginning of S-1 treatment and the date of death or last follow-up. Haematological toxicity, including neutropenia, leucopenia, thrombocytopenia and decreased haemoglobin level, was graded according to the National Cancer Institute Common Terminology Criteria for Adverse Events (NCI-CTCAE), version 3. The central data centre conducted onsite monitoring weekly during the first cycle and biweekly during and after the second cycle in order to confirm results of laboratory tests. The data centre collected and managed all the data regarding blood cell counts and grades of haematological toxicity.

### Statistical methods

In order to evaluate the prognostic implications of chemotherapy-induced neutropenia, we first identified the worst grade of neutropenia during treatment with S-1 for each patient. Owing to the size of the study group, we classified neutropenia into four categories: absent (grade 0), mild (grade 1), moderate (grade 2) and severe (grade 3–4). The survival curves of the four categories were estimated by the Kaplan–Meier method and compared by the log-rank test. This approach, considering the occurrence of myelosuppression during chemotherapy to be a baseline feature, has been used in many studies (Saarto *et al*, 1997; Poikonen *et al*, 1999; Cameron *et al*, 2003; Nakata *et al*, 2006) and is appropriate in an adjuvant setting, as the number of patients who die during the chemotherapy treatment period is usually negligible. In advanced cancer, however, considering myelosuppression to be a baseline feature can lead to a large bias because fewer cycles of chemotherapy can be administered due to poorer outcomes, consequently leading to a lower chance of neutropenia. In other words, patients who have better outcomes can receive many cycles of treatment, thus resulting in a higher incidence of chemotherapy-induced neutropenia. This can produce a false-positive association between chemotherapy-induced neutropenia and increased survival. To avoid this problem, a landmark analysis is sometimes employed, in which the study is limited to patients who survive for at least certain specific time period after the initiation of treatment, with neutropenia during that period considered as a baseline feature (Di Maio *et al*, 2005). However, such a landmark analysis would discard information regarding deaths occurring ‘before the landmark’, which could bias patient selection (Green *et al*, 2002). Further, our protocol required that treatment with S-1 would continue until the occurrence of disease progression or unacceptable toxicity. Consequently, a certain number of patients continued the treatment for prolonged period and experienced the late onset of neutropenia. A landmark analysis would easily be biased by neutropenia occurring ‘after the landmark’, and thus be unsuitable for our study. Instead, we treated chemotherapy-induced neutropenia as a time-dependent variable; for each patient, the worst grade of neutropenia occurring between the beginning of S-1 treatment and time *T*>0 was defined as the value of the variable at *T*. The variable value for each patient could change over time according to the worst grade of neutropenia experienced by that time. To quantify the impact of time-dependent neutropenia on survival, a Cox regression model was used to estimate the hazard ratio (HR) of death (Hosmer and Lemeshow, 1999). We also considered a multivariate Cox model that included other clinical features to obtain an adjusted HR and to examine the independent prognostic role of chemotherapy-induced neutropenia. A method by Simon and Makuch (1984) was used for a graphical representation of the survival curves according to the worst grade of neutropenia, which was considered to be a time-dependent variable; this method accounted for patients transferring from one group to another. All reported *P* values of statistical tests are two-tailed and *P*<0.05 was taken to be statistically significant. All analyses were performed using SAS (version 9.13) or S-PLUS (version 6.2).

## RESULTS

### Incidence of neutropenia

Table 1 shows the worst grade of neutropenia according to the treatment cycle of chemotherapy in the study subjects (*n*=1055). Cumulatively, a total of 637 cases of neutropenia (grades 1–4) were reported; among these, 206 cases were grade 1, 328 cases were grade 2, 91 cases were grade 3 and 12 cases were grade 4 (Table 1). The study protocol did not allow the prophylactic use of granulocyte-colony-stimulating factor (G-CSF), and no such use was reported. The results were therefore not biased regarding the use of G-CSF.

### Survival data according to the worst grade of neutropenia

The demographics and clinical/haematological characteristics of the study population are summarised in the leftmost column of Table 2. On the whole, patient demographics such as age, gender or BSA were very similar to those observed in the previous trials of S-1 (Sakata *et al*, 1998; Koizumi *et al*, 2000). The median values of the baseline leucocyte count and of the baseline neutrophil count were 6.3 × 10^9^ l^−1^ (range, 4.0–12.0) and 4.0 × 10^9^ l^−1^ (range, 2.0–10.9), respectively. The median RDI was 0.86 (range, 0.11–1.24), indicating that compliance for S-1 treatment was good overall.

There were 293 patients (28% out of the 1055 patients) who experienced neutropenia; among whom, 73 patients (7%) had grade 1 as the worst grade, 156 patients (15%) had grade 2 and 64 patients (6%) had grade 3 or higher. Of these patients with neutropenia, the worst grade initially occurred during the first cycle in 177 patients, during the second cycle in 57 patients, during the third cycle in 21 patients, during the fourth cycle in 19 patients, during the fifth cycle in patients and during the sixth or subsequent cycles in 11 patients. The characteristics of the patients, according to the worst grade of neutropenia experienced, are shown in Table 2. The dose intensity was lower in the patients with severe neutropenia; the median RDI in the patients with grade 3–4 neutropenia was 0.79 (range, 0.32–1.07), whereas that was 0.86 (range, 0.11–1.24) in the patients with no neutropenia (Table 2).

**Figure 1:**
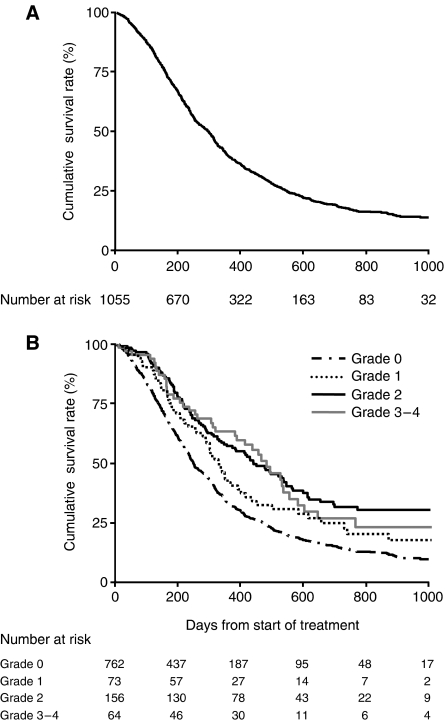
(**A**) Kaplan–Meier survival curve for all 1055 patients. (**B**) Kaplan–Meier survival curves according to the worst grade of chemotherapy-induced neutropenia. The log-rank test showed that the differences among the four curves were statistically significant (*P*<0.0001).

We compared the survival curves for subgroups of patients identified by the worst grade of neutropenia. As stated above, this simple approach may be subject to potential bias, but nevertheless it can be informative for a preliminary analysis. Figure 1 shows the survival curves (A) for all the patients and (B) for subgroups of patients stratified according to the worst grade of neutropenia. A total of 795 deaths (75%) were confirmed, including 767 deaths that were reported as tumour-related. The median follow-up of the 260 surviving patients was 472 days (range, 9–1136 days). The median survival of all the patients was 302 days (95% confidence interval (CI), 270–318 days) with 1- and 2-year survival rates of 40% (95% CI, 37–43%) and 18% (95% CI, 16–21%), respectively (Figure 1A). According to the severity of neutropenia, the median survival was 254 days (95% CI, 239–281 days) for patients with no neutropenia (grade 0), 355 days (95% CI, 309–415 days) for patients with mild neutropenia (grade 1), 459 days (95% CI, 377–559 days) for patients with moderate neutropenia (grade 2) and 480 days (95% CI, 380–552 days) for patients with severe neutropenia (grade 3–4) (Figure 1B). The log-rank test showed that the differences among the four curves were statistically significant (*P*<0.0001).

### Association of survival with the time-dependent neutropenia variable

**Figure 2:**
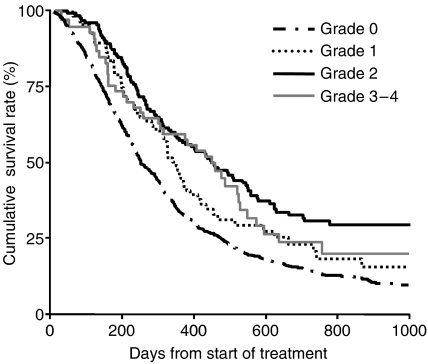
Survival curves as a function of chemotherapy-induced neutropenia treated in a time-dependent manner, by the method of Simon and Makuch with the landmark time of 14 days.

Table 3 shows the result of a Cox regression analysis for the association between overall survival and the worst grade of neutropenia, which was treated as a time-dependent variable. The HR for mild (grade 1) neutropenia in comparison with no neutropenia (grade 0) was 0.74 (95% CI, 0.56–0.98; *P*=0.0330), which translated into a 26% lower risk of death. Similarly, the HR for moderate (grade 2) neutropenia in comparison with no neutropenia was 0.55 (95% CI, 0.44–0.68; *P*<0.0001), which represented a 45% lower risk of death, and the HR for severe (grade 3–4) neutropenia in comparison with no neutropenia was 0.62 (95% CI, 0.44–0.85; *P*=0.0037), which was a 38% lower risk of death. Figure 2 shows the graphical representation of survival curves according to the worst grade of neutropenia in a time-dependent manner, allowing patients to be transferred from one group to another (Simon and Makuch, 1984; Richardson *et al*, 2003).

A multivariate Cox regression model including other clinical features (age, gender, BSA, ECOG PS, disease status, liver metastasis and leucocyte count) also demonstrated that any grade of neutropenia was independently associated with a better survival (Table 4). The adjusted HRs for mild, moderate and severe neutropenia in comparison with no neutropenia were 0.72 (95% CI, 0.54–0.95; *P*=0.0189), 0.63 (95% CI, 0.50–0.78; *P*<0.0001) and 0.71 (95% CI, 0.51–0.98; *P*=0.0388), respectively. Therefore, patients who experienced neutropenia had a more favourable prognosis, and the presence of moderate neutropenia suggested a higher efficacy of the drug than did the presence of either mild neutropenia or no neutropenia.

It is notable that patients with a leucocyte count ⩾9.0 × 10^9^ l^−1^ had a significantly poorer survival when compared with those with a leucocyte count <9.0 × 10^9^ l^−1^ (HR=1.46; 95% CI, 1.19–1.80; *P*=0.0004; Table 4). Several recent studies have reported that large numbers of leucocytes and their cytokine production correlate well with tumour development and severity (Coussens and Werb, 2002; Balkwill, 2004). It is also often clinically observed in advanced cancer that the neutrophil count elevates in accordance with an increased number of leucocytes. This implies that there is a potential link between a high density of neutrophils and a poor prognosis, thus leading to a false association between patients experiencing no neutropenia during chemotherapy and unfavourable survival outcomes. However, the multivariate analysis, adjusted by the leucocyte count, did not affect the significant association between neutropenia and survival, implying that neutropenia is independently predictive of the prognosis (Table 4).

We can observe that there was a trend for BSA being predictive of overall survival (HR=0.87; 95% CI=0.74–1.02; *P*=0.0878), although the statistical test did not reach a 5% significance (Table 4). This in turn suggested that higher dose might be related to better outcome in our dose-banding approach for S-1. However, BSA, which is defined by height and weight, showed a very high correlation with weight (Pearson correlation coefficient=0.962, *P*<0.0001) and it was thus likely that the above trend simply reflected the fact that patients maintaining weight tended to survive longer than those losing it. Hence, weight, which can be reasonably associated with patient’s general condition, could be a confounding factor in this case.

Finally, we performed similar analysis for other haematological manifestations of toxicity, including leucopenia, haemoglobin decrease and thrombocytopenia. However, none of these variables remained as significant factors in the multivariate model (data not shown).

## DISCUSSION

The objective of this study was to investigate whether chemotherapy-induced neutropenia was related to increased survival and could therefore be used as a surrogate indicator of patient prognosis. Since the late 1990s, several studies have linked myelosuppression induced by adjuvant chemotherapy to a better outcome in patients with breast cancer (Saarto *et al*, 1997; Poikonen *et al*, 1999; Mayers *et al*, 2001; Cameron *et al*, 2003). Recently, Di Maio *et al* (2005) reported the first evidence regarding the relationship between chemotherapy-induced neutropenia and longer survival time in patients with an advanced stage of cancer. They analysed the pooled data from three randomised trials of 1265 patients with advanced NSCLC treated with one of five different regimens, and concluded that both mild (grade 1–2) and severe (grade 3–4) neutropenia similarly predicted longer patient survival than did the absence of such toxicity. Nakata *et al* (2006) examined the prognostic role of neutropenia in a phase I study of 23 patients with advanced gastric cancer receiving S-1 plus cisplatin. These findings prompted us to perform a rigorous quantification of the prognostic value of chemotherapy-induced neutropenia with respect to survival outcomes in patients with advanced gastric cancer. In comparison with previous investigations, an advantage of our study is that a large number of patients received the same treatment with S-1 monotherapy according to the schedule used in previous clinical trials. Further, the survey entailed central data collection and management and frequent site-visit monitoring in order to ensure data quality.

In our study, the association between patients experiencing neutropenia and survival prolongation was considerable. The patients with a grade 1 or greater decrease in their neutrophil count had significantly better survival outcomes than did patients without such toxicity, indicating that chemotherapy-induced neutropenia may provide an index of delivering an optimal dose of chemotherapy that is required for generating an active antitumour effect. Moreover, the median RDI of S-1 in patients with neutropenia was less than that in patients with no neutropenia (Table 2). These results lead us to recognise the possibility that optimal dosing is not necessarily governed by the use of BSA-dosing guidelines; in fact, a poor correlation between BSA and the pharmacokinetics of most cytotoxic agents has been critically pointed out (Gurney, 1996, 2002; Ratain, 1998; Newell, 2002). Several prospective randomised studies have investigated the dose–response relationship in breast carcinoma (Fumoleau *et al*, 1993; Wood *et al*, 1994; Fisher *et al*, 1997) in order to assess the preference for higher doses than those guided by the BSA criteria. If, however, the BSA-based dosing system is not appropriate, then the optimal dose of chemotherapy for individual patients is relatively unrelated to whether the dose is high or low in terms of the BSA-based one.

When oncologists conduct phase II or III clinical trials, they predefine a schedule for dose reduction in the event of myelosuppression. In contrast, the dose is not increased in the absence of such toxicity. Similar guidelines are usually followed in daily clinical practice. This apparent asymmetry results from the presumption that the doses directed by BSA are valid for most, if not all, patients and that patients without toxicity such as myelosuppression will continue to derive substantial benefits from treatment. However, our study and that of others suggest that the absence of neutropenia may actually be a sign of an inadequate dose of chemotherapy (Kvinnsland, 1999). If this is the case, then the fact that more than 70% of patients in our study did not experience neutropenia in their treatment implies that the traditional BSA-based dose is far from optimal in ensuring that the majority of patients receive an effective dose.

Our analysis demonstrated that the patients with neutropenia had a significantly better survival than did the patients without neutropenia, and that severe (grade 3–4) neutropenia did not indicate a better survival than mild (grade 1) or moderate (grade 2) neutropenia. This result is precisely consistent with that obtained for the chemotherapy of NSCLC (Di Maio *et al*, 2005). In addition, our rigorous statistical approach elucidated that patients with moderate neutropenia reaped greater survival benefits from the treatment than did the patients with mild neutropenia. These findings provide us with a possible approach for fine-tuning of the initial dose of S-1, which is selected according to BSA; unless other severe toxicities are observed, a dose level that decreases the neutrophil count to 1.0–1.5 × 10^9^ l^−1^ (grade 2) is required for active antitumour effects. This approach would be most beneficial early on in treatment. Indeed, there were 884 (84% of the study population) patients who remained at grade 0–1 neutropenia in the first and second cycles of treatment. Of these, 428 (41%) patients experienced neither moderate/severe haematological toxicity (grade 2–4 leucopenia, haemoglobin decrease or thrombocytopenia) nor any grade of key non-haematological toxicity (grade 1–4 nausea, vomiting, diarrhoea, stomatitis, dermatosis) in the first and second cycles. An early dose increase aimed at moderate neutropenia could have been conducted safely for these patients and thus a significant number of patients were expected to fall under this category and receive more survival benefit from S-1.

On the other hand, a dose increase based on lack of neutropenia in the first and second cycles would clearly be a problem for patients exhibiting severe toxicity later on in treatment. In this sense, an early dose modification does not allow for the development of late toxicity. However, the number of patients who remained at grade 0–1 neutropenia in the first and second cycles but thereafter developed neutropenia or one of the above eight toxicities to a grade 3–4 level was only 54. Thus, an early dose increase would not be a problem for most of the patients targeted in our approach.

In our study, the prognostic impact of neutropenia as a marker for delivering the optimal dose was estimated to be 0.6–0.7 in terms of HR, similar to the survival benefit reported in the NSCLC study (Di Maio *et al*, 2005). This improvement in outcome would be equivalent to, if not greater than, that generally expected for chemotherapeutic regimens in experimental arms of phase III studies. Thus, the use of chemotherapy-induced neutropenia may prompt the more rational use of available drugs and benefit a large proportion of patients who are currently receiving unintentional under-dosing of cytotoxic chemotherapy. Although it remains unclear why the plateau of dose–response appears in proximity to the dose that produces modest neutropenia, accumulating evidence indicates that the possibility of using toxicity as a guide for tailored dosages deserves more attention for both curable and non-curable treatments.

In conclusion, we confirmed the prognostic implications of chemotherapy-induced neutropenia through the analysis of a large series of advanced gastric cancer patients treated with S-1. Our study suggests that chemotherapy-induced neutropenia can be used to individualise a pharmacologically active dose. Prospective randomised trials to explore safe intrapatient dose escalation with the intent of achieving neutropenia are thus warranted.

## Figures and Tables

**Table 1:** Worst grade of neutropenia according to the treatment cycle of chemotherapy

**Treatment cycle^a^**	**1**	**2**	**3**	**4**	**5**	**6**	**7**	**8**	**9–15^b^**	
**No. of patients**	**1055**	**813**	**520**	**398**	**296**	**224**	**169**	**132**	**104**	**Total**
Grade 1	63	44	31	25	17	5	5	6	10	206
Grade 2	108	66	48	39	21	15	8	7	16	328
Grade 3	31	22	12	10	4	4	4	3	1	91
Grade 4	6	3	1	1				1		12
										
Grade 1–4	208	135	92	75	42	24	17	17	27	637

a Single cycle consisted of 42 days (S-1 therapy for 28 consecutive days followed by 14 days of no treatment).

b Neutropenia was observed until the 15th cycle.

**Table 2:** Baseline demographics and clinical/haematological characteristics in all patients and in subgroups stratified according to the worst grade of neutropenia

	**All patients (*n*=1055)**	**Grade 0 (*n*=762)**	**Grade 1 (*n*=73)**	**Grade 2 (*n*=156)**	**Grade 3–4 (*n*=64)**
Age, median (range)	63 (23–80)	63 (23–80)	63 (28–78)	63 (34–80)	60 (26–80)
Gender, male/female (%)	768/287 (73/27)	574/188 (75/25)	48/25 (66/34)	105/51 (67/33)	41/23 (64/36)
BSA, median (range)	1.48 (1.04–2.01)	1.49 (1.11–2.01)	1.46 (1.04–1.88)	1.46 (1.08–1.87)	1.46 (1.10–1.74)
ECOG PS, 0/1 (%)	681/374 (65/35)	487/275 (64/36)	45/28 (62/38)	106/50 (68/32)	43/21 (67/33)
Disease status, recurrent/advanced (%)	341/714 (32/68)	244/518 (32/68)	21/52 (29/71)	46/110 (29/71)	30/34 (47/53)
Liver metastasis, absent/present (%)	775/280 (73/27)	530/232 (70/30)	56/232 (77/23)	134/22 (87/13)	55/9 (86/14)
Leucocytes, median (range)	6.3 (4.0–12.0)	6.6 (4.0–12.0)	6.0 (4.3–11.3)	5.5 (4.0–11.5)	5.6 (4.0–9.6)
Neutrophils, median (range)	4.0 (2.0–10.9)	4.3 (2.0–10.9)	3.9 (2.0–7.3)	3.4 (2.2–8.1)	3.3 (2.1–7.6)
Treatment cycles, median (range)	2 (1–20)	2 (1–18)	4 (1–14)	4 (1–20)	3 (1–16)
Treatment duration, median (range)	108 (2–889)	86 (2–889)	162 (28–609)	181 (7–794)	144 (3–754)
RDI, median (range)	0.86 (0.11–1.24)	0.86 (0.11–1.24)	0.85 (0.22–1.11)	0.85 (0.39–1.20)	0.79 (0.32–1.07)

Abbreviations: BSA, body surface area; ECOG PS, Eastern Cooperative Oncology Group performance status.

Units: age (years), BSA (m^2^), leucocytes ( × 10^9^ l^−1^), neutrophils ( × 10^9^ l^−1^), treatment duration (days).

**Table 3:** Cox model for the association between survival and chemotherapy-induced neutropenia^a^

**Neutropenia**	**Hazard ratio (95% CI)**	***P***
Grade 0	1.00 (referent)	
Grade 1	0.74 (0.56–0.98)	0.0330
Grade 2	0.55 (0.44–0.68)	<0.0001
Grade 3–4	0.62 (0.44–0.85)	0.0037

a Neutropenia is treated as a time-dependent variable.

**Table 4:** Multivariate Cox model for the association between survival and chemotherapy-induced neutropenia^a^

	**Adjusted hazard ratio (95% CI)**	***P***
*Neutropenia*		
1 *vs* 0	0.72 (0.54–0.95)	0.0189
2 *vs* 0	0.63 (0.50–0.78)	<0.0001
3–4 *vs* 0	0.71 (0.51–0.98)	0.0388
		
*Age*		
⩾60 *vs* <60	0.97 (0.84–1.13)	0.7012
		
*Gender*		
Male *vs* female	1.12 (0.94–1.34)	0.2167
		
*BSA (m^2^)*		
⩾1.50 *vs* <1.50	0.87 (0.74–1.02)	0.0878
		
*ECOG PS*		
1 *vs* 0	1.48 (1.28–1.71)	<0.0001
		
*Disease status*		
Advanced *vs* recurrent	1.20 (1.03–1.40)	0.0199
		
*Liver metastasis*		
Present *vs* absent	1.73 (1.47–2.03)	<0.0001
		
*Leucocytes ( × 10^9^ l* ^−*1*^ *)*		
9.0–12.0 *vs* 4.0–9.0	1.46 (1.19–1.80)	0.0004

Abbreviations: BSA, body surface area; ECOG PS, Eastern Cooperative Oncology Group performance.

a Neutropenia is treated as a time-dependent variable.
